# PREVALENCE OF LIFESTYLE-BASED MODIFIABLE DEMENTIA RISK FACTORS AMONG CANADIAN ADULTS AGES 50+

**DOI:** 10.1093/geroni/igae098.3401

**Published:** 2024-12-31

**Authors:** Danielle D’Amico, Brian Tan, Malcolm Binns, Howard Chertkow, Nicole Anderson

**Affiliations:** Baycrest Academy for Research and Education, Toronto, Ontario, Canada; Baycrest Academy for Research & Education, Toronto, Ontario, Canada; Baycrest Academy for Research & Education, Toronto, Ontario, Canada; Baycrest Academy for Research & Education, Toronto, Ontario, Canada; Baycrest Academy for Research & Education, Toronto, Ontario, Canada

## Abstract

Modifiable lifestyle behaviours are key determinants of cognitive health and dementia risk. The prevalence of lifestyle-based dementia risk factors is currently unknown among the Canadian adult population. The purpose of this study was to establish age- and sex-adjusted prevalence of risk in five domains using validated scales: 1) physical activity (CHAMPS); 2) brain-healthy eating (Eating Pattern Self-Assessment); 3) cognitive engagement (Florida Cognitive Activities Scale); 4) social connections (UCLA Loneliness Scale); and 5) mental well-being including depression, anxiety, distress, and perceived stress (DASS-21 and PSS-10). Males ages 70-79 (n=120) and females ages 60-69 (n=145), 70-79 (n=217), and 80+ (n=115) completed each scale online. Depending on the age decade x sex group, the percentage of individuals who exhibited mild depression, anxiety, distress, and perceived stress were 20.0-28.7%, 14.7-22.6%, 13.0-20.0%, and 43.3-54.7%, respectively. 33.1-54.5% did not meet the physical activity recommendations of 150 minutes/week of moderate to vigorous exercise. No participants met the brain-healthy eating recommendations. High-risk cut-off scores (below the 10th percentile) ranged from 8.0-11.6 for cognitive engagement and 23.4-24.4 for social engagement. The number of domains upon which individuals were at high risk ranged from 1 to 5 (median = 4). Data are currently being collected for females ages 50-59 and males ages 50-69 and 80+. This study is part of the first-of-its-kind research-based community centre at Baycrest delivering personalized dementia risk reduction programming. These data will be used to identify domains in which participants are at-risk in order to guide personalized dementia risk reduction program strategy.

